# Long-Term Vegetation Dynamics in a Megadiverse Hotspot: The Ice-Age Record of a Pre-montane Forest of Central Ecuador

**DOI:** 10.3389/fpls.2018.00196

**Published:** 2018-02-20

**Authors:** Encarni Montoya, Hayley F. Keen, Carmen X. Luzuriaga, William D. Gosling

**Affiliations:** ^1^School of Environment, Earth and Ecosystem Sciences, The Open University, Milton Keynes, United Kingdom; ^2^Instituto de Ciencias de la Tierra Jaume Almera, Consejo Superior de Investigaciones Científicas, Barcelona, Spain; ^3^Estación Biológica de Pindo-Mirador, Universidad Tecnológica Equinoccial, Quito, Ecuador; ^4^Institute for Biodiversity and Ecosystem Dynamics, University of Amsterdam, Amsterdam, Netherlands

**Keywords:** diversity dynamics, eastern Andean flank, Last Glacial Maximum, neotropics, palaeoecology, stability, vulnerability, western equatorial Amazonia

## Abstract

Tropical ecosystems play a key role in many aspects of Earth system dynamics currently of global concern, including carbon sequestration and biodiversity. To accurately understand complex tropical systems it is necessary to parameterise key ecological aspects, such as rates of change (RoC), species turnover, dynamism, resilience, or stability. To obtain a long-term (>50 years) perspective on these ecological aspects we must turn to the fossil record. However, compared to temperate zones, collecting continuous sedimentary archives in the lowland tropics is often difficult due to the active landscape processes, with potentially frequent volcanic, tectonic, and/or fluvial events confounding sediment deposition, preservation, and recovery. Consequently, the nature, and drivers, of vegetation dynamics during the last glacial are barely known from many non-montane tropical landscapes. One of the first lowland Amazonian locations from which palaeoecological data were obtained was an outcrop near Mera (Ecuador). Mera was discovered, and analysed, by Paul Colinvaux in the 1980s, but his interpretation of the data as indicative of a forested glacial period were criticised based on the ecology and age control. Here we present new palaeoecological data from a lake located less than 10 km away from Mera. Sediment cores raised from Laguna Pindo (1250 masl; 1°27′S, 78°05′W) have been shown to span the late last glacial period [50–13 cal kyr BP (calibrated kiloyears before present)]. The palaeoecological information obtained from Laguna Pindo indicate that the region was characterised by a relatively stable plant community, formed by taxa nowadays common at both mid and high elevations. *Miconia* was the dominant taxon until around 30 cal kyr BP, when it was replaced by *Hedyosmum*, Asteraceae and *Ilex* among other taxa. Heat intolerant taxa including *Podocarpus*, *Alnus*, and *Myrica* peaked around the onset of the Last Glacial Maximum (c. 21 cal kyr BP). The results obtained from Laguna Pindo support Colinvaux’s hypothesis that glacial cooling resulted in a reshuffling of taxa in the region but did not lead to a loss of the forest structure. Wide tolerances of the plant species occurring to glacial temperature range and cloud formation have been suggested to explain Pindo forest stability. This scenario is radically different than the present situation, so vulnerability of the tropical pre-montane forest is highlighted to be increased in the next decades.

## Introduction

The degree to which the structure and composition of vegetation in tropical South America has been altered in response to high magnitude past global climate change has been long debated (Haffer, 1969; Liu and Colinvaux, 1985; Bush et al., 1990; Absy et al., 1991; Heine, 1994). Revealing the sensitivity of tropical forests to past climate change is the only way in which empirical data can be obtained into how this complex biodiverse region is likely to respond to projected future climate change (Cox et al., 2000; Myers et al., 2000; Malhi and Wright, 2004; IPCC, 2013). Furthermore, it is only by exploring the fossil record that we can parameterise the speed of change that the vegetation has experienced with in the past and consequently gain an idea of the rate at which in may be able to change in the future. Palaeoecology contains powerful tools, such as fossil pollen analysis, with which the dynamics of the vegetation communities through time can be unravelled (Von Post, 1916). The global Last Glacial Maximum (LGM) period (26.5–19 kyr BP; Clark et al., 2009) saw temperatures in the South American tropics of between 4 and 5°C and up to 8°C cooler than modern in the Andes (Bush et al., 2007), and 4–7°C cooler than the modern Amazon (Liu and Colinvaux, 1985; Bush et al., 1990). The LGM-to-modern warming to which tropical South America was subject to over the past 20 ka is, therefore, equivalent to the projected magnitude of change for the next century (IPCC, 2013). In addition, during the last glacial period precipitation (Mosblech et al., 2012) and landscape processes (Loughlin et al., 2018) are likely to have contributed to vegetation change. However, due to a paucity of study sites little is known about the structure and composition of tropical South American glacial vegetation and how it changed during the last glacial period (Colinvaux et al., 1996; Flantua et al., 2015).

The first evidence of glacial vegetation obtained through fossil pollen analysis comes from an outcrop on the Ecuadorian eastern Andean flank near the town of Mera (Liu and Colinvaux, 1985). The glacial sediments from the Mera section were interpreted as containing fossils from a mixture of lowland vegetation and other taxa that live nowadays at higher elevations, and were used to infer a temperature decrease of around 4.5°C compared to present-day. These data were received with scepticism by some researchers, especially regarding: (1) the chronology of the section (Heine, 1994), and (2) the tolerance range of ecological conditions of some of the taxa identified in the record (Gentry, 1993). In fact, some of these debates are still ongoing (Cárdenas et al., 2011a,b; Puyasena et al., 2011). The use of outcrops represents the primary source of sedimentary archives in a very geomorphologically active region (Hall et al., 2008; Lombardo, 2014, 2016). However, outcrops often only represent a short time window and do not contain sediments extending up to the present so consequently interpretation can sometimes be challenging. In addition, outcrops on the eastern Andean flank normally contain interbedded layers of organic (pollen-rich) and inorganic (tephra-like) layers (Cárdenas et al., 2014; Loughlin et al., 2018). This mixture of processes that lead to the sediments’ deposits can easily compromise the continuity of the record by containing numerous sedimentary gaps or hiatuses between the different layers, preventing the study of the vegetation changes in a continuous, dynamic fashion.

Here we present for the first time a continuous vegetation dynamics record from a lacustrine sequence of a mid-elevation Ecuadorian forest located within the diversity hotspot of the eastern Andean flank, in western Amazonia. The glacial dynamics will be explored based on pollen analysis, and supported by charcoal and stable isotope analyses. The aim is to reconstruct the full to late glacial vegetation dynamics, from around 50 to 13 cal kyr BP (calibrated kiloyears before present), as well as to derive potential palaeoclimatic and palaeoecological inferences. The study focuses on the potential changes that might have occurred around the LGM, and it was prompted by the lack of glacial lacustrine palaeoecological studies in the region and the unknown responses of its unique and endangered ecosystem to potential environmental changes. Emphasis will be placed on diversity and stability dynamics, as well as to identify the drivers that have triggered such dynamism. Final details about the sensitivity and resilience of the glacial forest that preceded the current plant community as well as the nature of the forest components will be discussed.

## Materials and Methods

### Study Area

**FIGURE 1:**
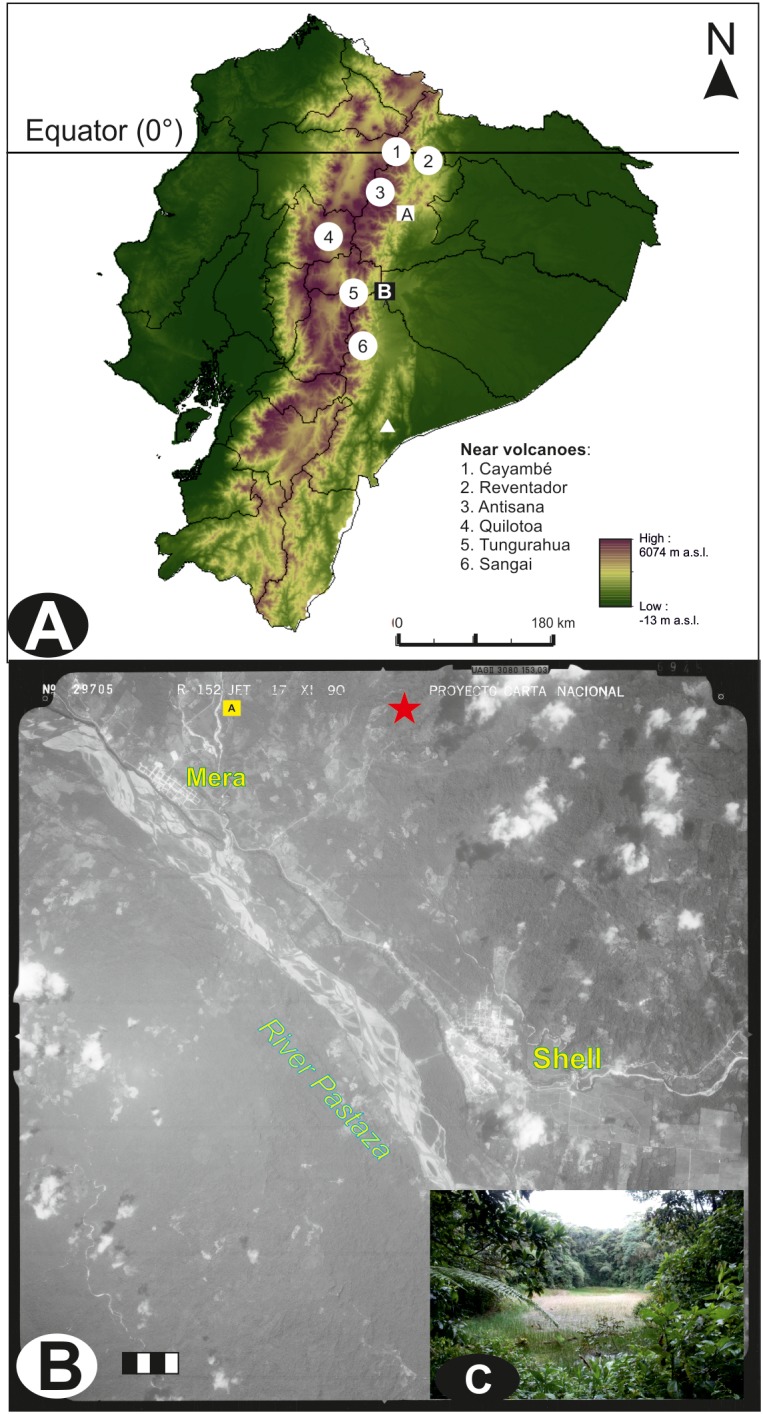
Study area. **(A)** Map of Ecuador, showing the location, main Quaternary active volcanoes near the study site (circles), the speleothem Santiago record (triangle) and other sedimentary archives mentioned in the text (squares): (A) Vinillos (Loughlin et al., 2018), and (B) Mera and Laguna Pindo (the present study). **(B)** Aerial photograph of the specific study area showing the detailed location and proximity of Laguna Pindo (star) and the closest sequence Mera (square). Main geographical features including river (italics) and towns (bold) are also shown. Year of the photograph: 1990; scale 1:60,000 (bar = 1 km). Photograph obtained from the Ecuadorian Geographic Military Institute (IGM). **(C)** Picture of the lake where it can be observed the closed vegetation surrounding the lake’s edge (February 2013).

Laguna Pindo (1°27′S–78°05′W) is a small shallow lake (c. 1.2 m depth), roughly circular shaped (c. 40 m diameter), located in the Pastaza province near the town of Mera at an elevation of 1248 masl (**Figure 1**). Mean annual temperature is about 20.8°C with little seasonal variation, annual precipitation can reach up to 4800 mm per year (Ferdon, 1950; Hijmans et al., 2005). Currently the lake is not directly fed by an in-flow and has no visible out-flow; the lake receives water from surface run-off and via direct precipitation, with a rough estimation of a small catchment of around 2–3 km (**Figure 1**). The study site is positioned in the Andean foothills on a steep slope dropping down to the Pastaza river basin (**Figure 1**), there are no obvious geomorphological causes for the escarpment of the lake and we hypothesise it is tectonic in origin (Matthews-Bird et al., 2017). The underlying geology of the eastern Andean flank is composed primarily of metamorphic rocks of Palaeozoic to Jurassic age (Aspden and Litherland, 1992; de Berc et al., 2005). The rocks were metamorphosed during the late Cretaceous and the Paleocene, and subsequently were overlain by volcanic/volcaniclastic formations of late Miocene to Quaternary origin (Bernal et al., 2011, 2012). Specifically, the inorganic sediment recovered at the bottom of the sequence has been classified as basalt (Matthews-Bird et al., 2017). Laguna Pindo is a mid-elevation site at the transition between the high elevation *páramo* vegetation and lowland Amazonia rain forest. The site lies within an area classified as lower montane rain forest (Harling, 1979), or pre-montane forest (Sierra, 1999), just below the lower limit of the cloud forest. The lake is in an advanced stage of filling in, with abundant aquatic plants (*Eleocharis maculosa*, Cyperaceae) growing within the lake. The lake is completely surrounded by a closed belt of vegetation to the water’s edge. The closed forest surrounding the site has a canopy of 15–25 m high; the dominant species belong to families of Melastomataceae, Araceae, Cecropiaceae, Euphorbiaceae, Myrtaceae, Rubiaceae, Myristicaceae, Asteraceae, and Mimosaceae. Lianas, epiphytes (Bromeliaceae, Orchidaceae), and tree ferns are also common (Jørgensen and León-Yánez, 1999). A survey of the vegetation belt at the lake’s edge is presented in **Table 1**. The lake is remote and currently beyond the influence of direct human activity such as agriculture and urbanisation, except for the presence of a biological station nearby.

**Table 1:** List of main vegetation taxa currently present surrounding Laguna Pindo based on rough field survey by C. X. Luzuriaga in 2013 and Luzuriaga (2007).

Taxon	Family	Type
*Alchornea leptogyna*	Euphorbiaceae	Tree
*Aniba hostmanniana*	Lauracea	Tree
*Anthurium* sp.	Araceae	Epiphyte
*Cabralea canjerana*	Meliaceae	Tree
*Calathea lutea*	Maranthaceae	Herb
*Cecropia engleriana*	Cecropiaceae	Tree
*Ceiba pentandra*	Bombacaceae	Tree
*Celtis guianensis*	Ulmaceae	Tree
*Clusia pallida*	Clusiaceae	Hemiepiphyte
*Cordia alliodora*	Boraginaceae	Tree
*Costus amazonicus*	Costaceae	Herb
*Croton lechleri*	Euphorbiaceae	Tree
*Dacryodes olivifera*	Burseraceae	Tree
*Eugenia* cf. *dibrachiata*	Myrtaceae	Tree
*Guadua angustifolia*	Poaceae	Tree
*Heliconia stricta*	Heliconiaceae	Herb
*Inga silanchensis*	Mimosaceae	Tree
*Inga velutina*	Mimosaceae	Tree
*Laetia procera*	Flacourtiaceae	Tree
*Macrolobium acaciifolium*	Caesalpiniaceae	Tree
*Matisia cordata*	Bombacaceae	Tree
*Miconia barbeyana*	Melastomataceae	Shrub–treelet–tree
*Miconia dielsi*	Melastomataceae	Shrub–tree
*Miconia splendens*	Melastomataceae	Shrub–treelet–tree
*Miconia* sp.	Melastomataceae	Shrub–treelet–tree
*Nectandra coeloclada*	Lauracea	Tree
*Ocotea cernua*	Lauracea	Tree
*Otoba parviflora*	Myristicaceae	Tree
*Palicourea guianensis*	Rubiaceae	Tree
*Piper aduncum*	Piperaceae	Shrub–treelet
*Pollalesta discolor*	Asteraceae	Tree
*Pourouma guianensis*	Cecropiaceae	Tree
*Pouteria multiflora*	Sapotaceae	Tree
*Sapium marmieri*	Euphorbiaceae	Tree
*Senna ruiziana*	Caesalpiniaceae	Tree
*Siparuna schimpffii*	Monimiaceae	Shrub–tree
*Socratea exorrhiza*	Arecaceae	Tree
*Saurauia prainiana*	Actinidiaceae	Shrub–treelet
*Syzygium jambos*	Myrtaceae	Tree
*Trema micrantha-t*^∗^	Ulmaceae	Tree
*Turpinia occidentalis*	Staphyleaceae	Tree
*Viburnum ayavacense*	Caprifoliaceae	Shrub
*Vismia baccifera*	Clusiaceae	Tree
*Vochysia braceliniae*	Vochysiaceae	Tree
*Wettinia maynensis*	Arecaceae	Tree
*Xanthosoma* sp.	Araceae	Herb
*Zanthoxylum kellermani*	Rutaceae	Tree

*Family and plant type have been included following Jørgensen and León-Yánez (1999). Asterisk “^∗^” refers to visual ID in field only by common name (e.g., Sapán negro), caution must be taken.*

### Methodology

A sediment core was extracted from the deepest point of the lake in January 2013 using a cam-modified Livingstone piston corer (Livingstone, 1955; Colinvaux et al., 1999). The sediment core recovered had a total length of 924 cm. This study presents a multi-proxy investigation (sediment characteristics, pollen, charcoal, and stable isotope) of the lower section of the sediment core (514–924 cm), which has been dated to the last glacial period. Eighteen samples were selected through the entire sequence and sent to the NERC Radiocarbon Facility, SUERC, East Kilbride, Scotland for radiocarbon analysis by accelerator mass spectrometery (**Table 2**). An age-depth model was constructed using the statistical package “clam” in R (Blaauw, 2010) using the calibration curve SHCal.13.14c (Hogg et al., 2013).

**Table 2:** Conventional (yr BP) and calibrated (cal kyr BP) radiocarbon data used in construction of chronologies for Laguna Pindo.

Publication code	Depth (cm)	δ^13^C_V PDB_ (‰)^∗^	^14^C age (yr BP)	Calendar age (cal kyr BP) 2σ
SUERC-54395^b^	46	-30.2	334 ± 42	289–470
SUERC-47634^b^	117	-27.9	974 ± 36	769–923
SUERC-47635^b^	245	-27.3	1973 ± 39	1812–1943
SUERC-47569^b^	329	-24.9	2335 ± 37	2293–2361
SUERC-47572^b^	410	-22.7	2829 ± 39	2781–2991
SUERC-48854^a^	461	-28.7	3974 ± 45	4241–4447
SUERC-54385^a^	483	-27.9	4518 ± 40	4969–5300
SUERC-54386^a^	504	-27.8	5641 ± 39	6298–6454
SUERC-54387^a^	510	-27.4	6029 ± 42	6717–6946
SUERC-61456^a^	512	-28.4	7897 ± 41	8542–8784
SUERC-61457^a^	514	-28.1	11,697 ± 46	13,387–13,580
SUERC-61458^a^	517	-28.0	13,945 ± 54	16,581–17,073
SUERC-48855^a^	521	-28.0	13,982 ± 59	16,618–17,138
SUERC-54388^a^	551	-28.1	20,153 ± 104	23,907–24,450
SUERC-61459^a^	579	-29.5	28,332 ± 284	31,449–32,998
SUERC-45933^b^	605	-23.6	33,417 ± 519	36,354–38,781
SUERC-61505^a^	660	-29.2	39,406 ± 1086	41,942–45,129
SUERC-56825^b^	725	-26.7	43,425 ± 1788	44,725–49,907

*^∗^δ^13^C values were measured on a dual inlet stable isotope mass spectrometer (Thermo Scientific Delta V Plus) and are representative of δ^13^C in the pre-treated sample material. ^a^Bulk sediment samples; ^b^wood remains’ samples. Samples were 1 cm thick.*

Samples for pollen analysis (1 cc of wet sediment and 1 cm thickness) were processed using standard methods including KOH, HCl, and HF digestions, acetolysis and mounting/storing in glycerin jelly (Faegri and Iversen, 1989). *Lycopodium* tablets (University of Lund batch n° 124961; 12,542 spores/tablet) were added before chemical processing (Stockmarr, 1971). Counting was conducted until a minimum of 300 pollen and spores and the saturation of diversity (Rull, 1987). The pollen sum included all pollen types with the exception of aquatic plants (Cyperaceae, *Myriophyllum*, *Sagittaria*, *Utricularia*). Identification was based on the reference collection held at The Open University (United Kingdom), and regional floras and atlases (e.g., Roubik and Moreno, 1991; Colinvaux et al., 1999; Bush and Weng, 2006). Given the diversity of the study area, non-identified morphotypes were coded with the acronym UPP (Unidentified Pollen grain from Pindo). Charcoal particles were identified and counted in the same palynological slides, only particles >5 μm were considered and two different classes were established based on size: (1) small particles (>5–100 μm), indicative of regional fires due to easy dispersion by wind, and (2) big particles (>100 μm), indicative of fires occurred more in a local scale (Whitlock and Larsen, 2001).

Analysis of the stable isotopes δ^13^C and δ^15^N was performed at a 4–10 cm sampling interval. Samples for δ^13^C and δ^15^N were obtained from ∼0.6 g sample aliquots that were homogenised and treated sequentially with 0.1 and 1 M HCl for 24 h, before being rinsed to neutrality with Milli-Q water (18.2 MΩ cm^-1^). Each step, involving a change of reagent or water, was preceded by centrifugation to prevent the loss of fine material in suspension. The isotopic composition (δ^13^C and δ^15^N) of the dried re-homogenised residues was then determined using a Thermo Flash HT elemental analyser equipped with a Thermo zero-blank device coupled to a Thermo MAT 253 mass spectrometer (EA-MS). Results are expressed following the guidelines for the reporting of stable isotope measurement results (Coplen, 2011).

Pollen diagram, diversity measures, and cluster analyses were performed in R version 3.82 using the packages “vegan” 2.3-5 (Oksanen et al., 2013) and “rioja” (Juggins, 2017). For the cluster analysis the dataset used was the percentage data after square root transformation and the cluster method used was “average” (after calculating the dissimilarity). Zonation was obtained by CONISS using the broken stick method to determine the significant zones (Bennett, 1996). RoC as defined by Urrego et al. (2009) were calculated using the R package PaleoMas (Correa-Metrio et al., 2010). Diversity measures include N_0_ (richness or species number, also called S), N_1_ and N_2_, calculated following Hill (1973). For calculating the indices, the dataset used was the raw data without downweight of rare taxa in order to capture the total diversity values.

## Results

### Sediment Description and Chronology

**FIGURE 2:**
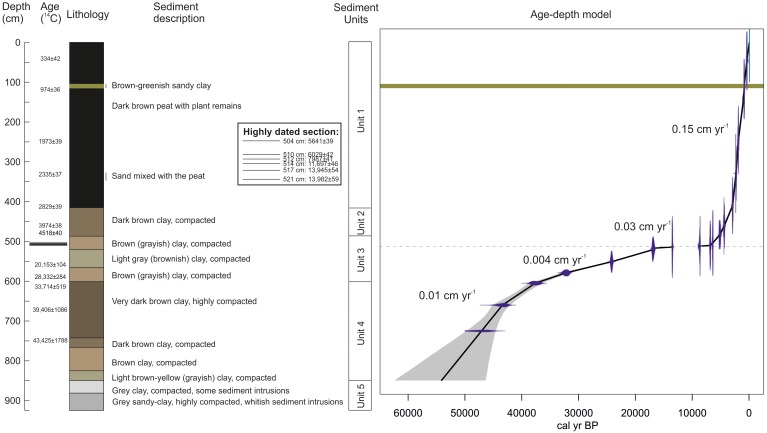
Simplified sediment description, age-depth model and accumulation rates calculated. Age-depth model was calculated only considering the organic sediment where proxies were preserved. Dash line in the age-depth model marks the position of the hiatus, and the green band represents an Holocene tephra.

The sediment recovered from Laguna Pindo consists mostly of peat and clay with different levels of organic content, and frequent wood remains interbedded in the sediment. Based on the differences found, five sedimentary units were defined (**Figure 2** and **Table 3**). The glacial interval was found in the sedimentary record from 514 cm downwards, corresponding to the Units 4 and 5, and the bottom section of Unit 3, delimited by the presence of a hiatus (**Figure 2**).

**Table 3:** Sedimentary units defined for Laguna Pindo core including main features.

Unit	Depth (cm)	Sediment	Colour	Features
Unit 1	0–414	Organic peat	10YR-2/2	The interval between 182 and 307 cm is characterised by numerous large wood remains
				Within this interval, a tephra is preserved at 105–114 cm depth (colour: 2.5YR-2/2), dated around 850 years ago and likely originated from Tungurahua or Quilotoa volcanoes event (Matthews-Bird et al., 2017)
Unit 2	414–482	Organic clay	2.5YR-3/3	Gradual change to upper unit (Unit 1)
			2.5YR-3/1	
Unit 3	482–601	Light clay	2.5YR-4/2	Very gradual transition colours, from darker to the extremes to lighter in the medial zone. Sediment compacted
			2.5YR-5/2	
			2.5YR-6/2	
			2.5YR-5/4	
Unit 4	601–850	Organic clay	10YR-2/2	Compacted sediment with occasional large wood remains
			10YR-3/2	
			10YR-4/2	
			10YR-3/2	
			10YR-4/2	
Unit 5	850–924	Inorganic clay	2.5YR-5/2	Highly compacted. XRF analysis of major elements located this sediment in a TAS diagram within the basalt domain (Matthews-Bird et al., 2017)
			2.5YR-6/1	
			2.5YR-7/2	
			2.5YR-6/1	
			2.5YR-8/3	

*Munsell Colour Chart was used for defining the sediment colours.*

An age-depth model for Laguna Pindo was constructed in Clam.R based on eighteen radiocarbon dates samples (**Table 2**). The best fit was obtained with a linear interpolation (**Figure 2**), allowing the calculation of the RoC following Urrego et al. (2009) (**Table 4**). The sedimentation rate was found to be highly variable, ranging from 0.0009 to 0.25 cm yr^-1^, with an average of 0.074 cm yr^-1^ (**Figure 2**). For the glacial interval, the sedimentation rate found was at least an order of magnitude slower compared to the Holocene section, ranging between 0.02 and 0.003 cm yr^-1^ for most of the interval, and showing the lowest value of 0.0009 cm yr^-1^ for the upper part of the glacial section (late Glacial).

**Table 4:** Ecological metrics of Laguna Pindo glacial vegetation based on pollen data: rates of change (RoC) were calculated following Urrego et al. (2009), and diversity indices (N_0_, N_1_, N_2_, and the ratio N_2_/N_0_) following Hill (1973).

Age	RoC	N_0_	N_1_	N_2_	N_2_/N_0_
13,487	NA	57	23.091	13.71	0.24
15,685	1.21 × 10^-4^	45	24.697	17.54	0.39
16,783	4.03 × 10^-4^	52	24.738	15.76	0.30
18,402	1.62 × 10^-4^	47	26.729	18.25	0.39
20,809	1.91 × 10^-4^	45	19.806	12.75	0.28
22,975	1.05 × 10^-4^	51	23.592	14.97	0.29
25,614	1.15 × 10^-4^	43	20.640	13.96	0.32
28,486	3.82 × 10^-5^	50	22.840	14.36	0.29
31,358	1.51 × 10^-4^	50	20.729	12.92	0.26
33,673	4.69 × 10^-5^	50	19.781	11.42	0.23
40,875	5.48 × 10^-5^	45	16.710	7.71	0.17
44,868	7.68 × 10^-5^	50	23.269	14.07	0.28
47,135	9.18 × 10^-5^	50	17.738	7.50	0.15
50,534	5.83 × 10^-5^	56	16.611	7.52	0.13
52,574	3.77 × 10^-4^	40	18.887	11.17	0.28

*Sample age is reported in cal kyr BP.*

### Pollen Zones

**FIGURE 3:**
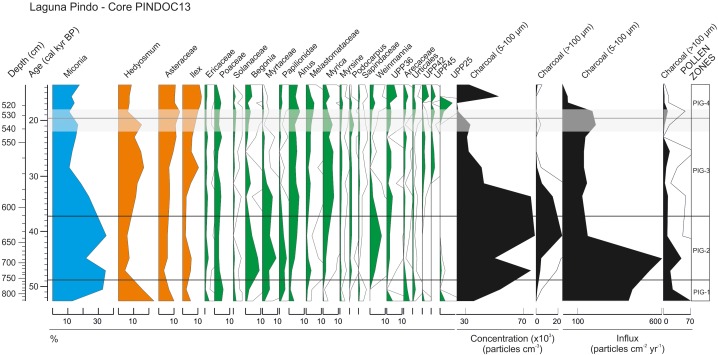
Pollen diagram of the glacial sequence of Laguna Pindo expressed in percentages, including microcharcoal particles results expressed both in concentration (particles cm^-3^) and influx (particles cm^-2^ yr^-1^) values. All the taxa percentages are shown with the same scale (equal width means equal % values) and outline values represent ×10 exaggeration. The grey shade shows the LGM core interval (22–18 cal kyr BP). Taxa have been grouped and coloured following **Figure 4**.

Pollen grains were found in Laguna Pindo until 822 cm depth, coinciding with the beginning of the sedimentary Unit 5 (**Figure 2**), which was barren for any biological remain. The pollen diagram of Laguna Pindo during the glacial interval delimits four significant zones based on differences in the most abundant taxa, only taxa occurring at percentages higher than 10% are represented (**Figure 3**). Charcoal particles have been calculated both as concentration and influx values. The two orders of magnitude difference in the sedimentation rate between the top and bottom of the sequence is generating an artefact in the charcoal influx curve, which is masking the values attained at the top of the sequence (**Figure 3**). In this sense, results will be described following the concentration curve, although the interpretation of both curves will be provided in the next section.

#### Pollen Zone PIG-1: From 822 to 756 cm; >50–48.8 cal kyr BP

The oldest section of Laguna Pindo is marked by a decrease to the top of the zone of *Hedyosmum* and Asteraceae, and in a minor extent *Alnus* and some unidentified morphotypes such as UPP36, UPP42, and UPP25. At the same time, *Miconia* shows the opposite trend. Total pollen concentration ranges from 257,000 to 1,000,000 pollen grains cc^-1^. The charcoal record during this interval is low (**Figure 3**).

#### Pollen Zone PIG-2: From 756 to 611 cm; 48.8–37.3 cal kyr BP

PIG-2 shows the inverse relationship between the dominant taxa of the previous zone *Hedyosmum* and *Miconia*, with low and high values, respectively (**Figure 3**). *Weinmannia* appears during this zone and *Alnus*, *Myrica*, *Myrsine*, *Podocarpus*, and UPP36 increase the abundance attained in the previous zone. On the contrary, taxa including Sapindaceae, Urticales, and unidentified UPP42 and UPP25 greatly decrease or disappears from the record. *Begonia*, *Ilex*, and Melastomataceae are also abundant at the beginning of the zone, but start decreasing towards the upper section. This zone is characterised in the upper half by the maximum values of charcoal, both small and big size (indicative of regional and local fires, respectively). Regarding pollen concentration, the values in this zone are also the highest of the record ranging from 436,000 to 1,279,000 pollen grains cc^-1^.

#### Pollen Zone PIG-3: From 611 to 532 cm; 37.3–19.6 cal kyr BP

The most dramatic change of the vegetation surrounding Pindo during glacial time corresponds to the decrease observed in *Miconia* during this zone to values below 20%, until the middle of the section. Following the previous zone, *Hedyosmum* shows an opposite character to *Miconia*, evident in this zone with a subtle but solid increase. Coeval to the minimum value of *Miconia*, a peak in *Ilex* is observed, followed by a steadier increase towards the top of the zone. UPP42 and UPP45 reappear during this zone, whereas Papilionidae, *Weinmannia*, and Myrtaceae decrease. The charcoal curve follows the high values attained at the end of the previous zone until approximately 32 cal kyr BP when they suddenly decrease to half of the particles abundance. Total pollen concentration in PIG-3 ranges from 277,000 to 905,000 pollen grains cc^-1^.

#### Pollen Zone PIG-4: From 532 to 514 cm; 19.6–13.5 cal kyr BP

The post-LGM and late Glacial interval of Laguna Pindo is characterised by low values of both *Hedyosmum* and *Miconia* compared to the rest of the sequence. UPP25 peaks at the beginning of the zone and disappears again. Asteraceae and *Ilex* attain during this zone their highest values, and Myrtaceae shows its lowest abundance. Charcoal particles present in this zone are among the minimum values of the entire sequence, and the same occurs with the pollen concentration, ranging from 211,000 to 544,000 pollen grains cc^-1^.

### Additional Metrics for Plant Dynamics

**FIGURE 4:**
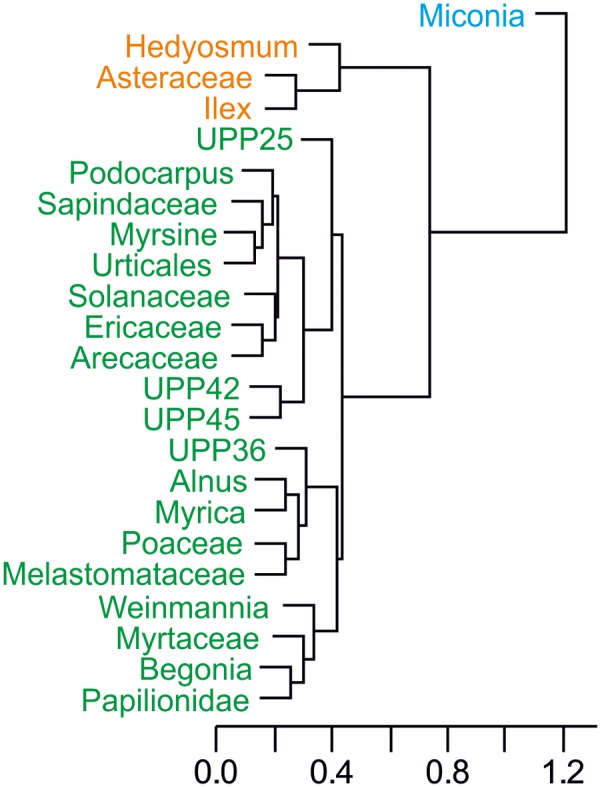
Cluster analysis through the “average” method of the most abundant pollen taxa found in Laguna Pindo during the glacial interval. Colours define the groups resulted: Group 1 (blue), Group 2 (orange), and Group 3 (green).

**FIGURE 5:**
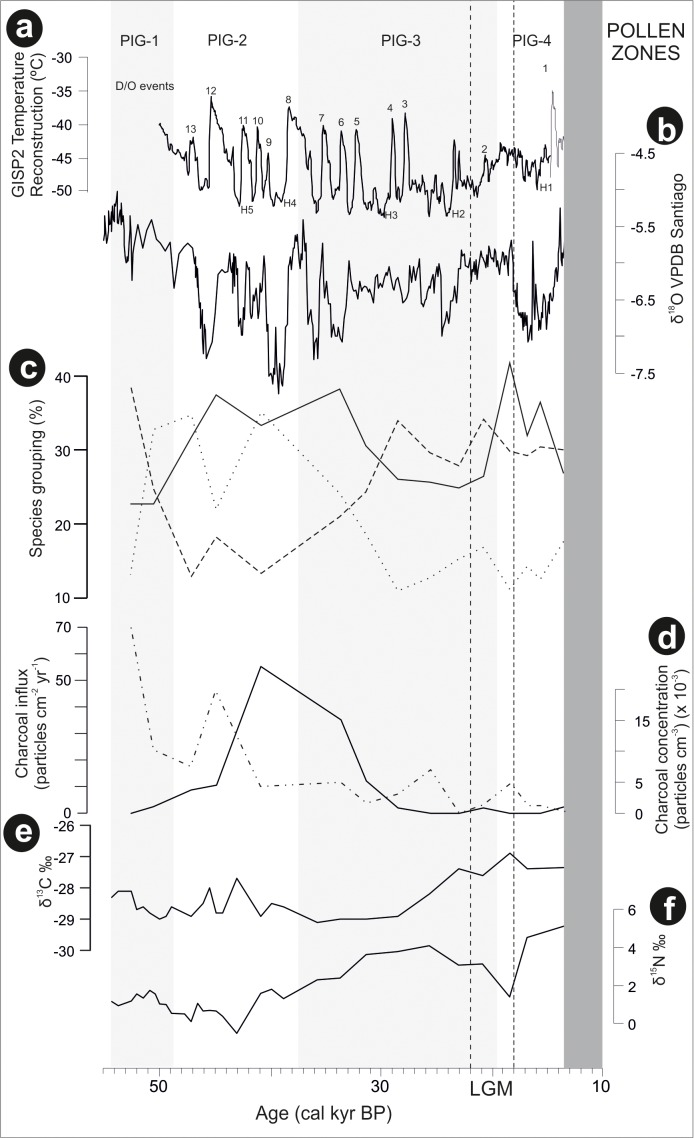
Laguna Pindo metrics and additional data framed in a regional palaeoclimatic context (raw data downloaded from NOAA): **(a)** GISP2 temperature reconstruction of Greenland (Alley, 2000), in which are shown the Heinrich (H) and Dansgaard–Oeschger (D/O) events that occurred in the time interval under study; **(b)** precipitation reconstruction based on Santiago speleothem record (Mosblech et al., 2012); **(c)** percentages of the groups resulted in the cluster analysis of Laguna Pindo most abundant palynological taxa (**Figure 4**): with dotted, dashed, and continuous lines showing the different groups (Group 1, 2, and 3 of **Figure 4**, respectively); **(d)** local fires curve of Laguna Pindo based on big (>100 μm) charcoal particles, expressed in concentration and influx values (continuous and discontinuous lines, respectively); **(e)** δ^13^C of Laguna Pindo, expressed in ‰; and **(f)** δ^15^N of Laguna Pindo, expressed in ‰. The dark grey shade represents the hiatus of Laguna Pindo, calculated as the calibrated ages of the dated samples bracketing the sedimentary gap (at 512 and 514 cm depth), and the light grey bands mark the pollen zones obtained. Vertical dashed lines show the LGM core interval following **Figure 3**.

Several tests were also run to get a better idea of the palynological dynamics. A cluster analysis of the represented taxa (percentages above 10%) was performed to see the grouping formed by the most abundant pollen morphotypes with similar distributions along the sequence (**Figure 4**). The plot shows three different groups well defined, with only *Miconia* belonging to the first group; *Hedyosmum*, Asteraceae, and *Ilex* forming the second group; and the rest of morphotypes (19) included in Group 3. Stable isotopes of C and N were plotted stratigraphically against age (**Figure 5**), providing values between -30 and -25‰ for δ^13^C, indicative of mostly C3 land plants, and between 0 and 5‰ for δ^15^N, representing a mixed primary production source formed by aquatic and terrestrial plants (Meyers and Teranes, 2001). δ^13^C sees an increasing trend starting around 25 cal kyr BP. δ^15^N shown an earlier increasing trend around 43 cal kyr BP including a brief drop around 19 cal kyr BP. Finally, some diversity metrics were calculated following Hill (1973), as previous works have highlighted their suitability for pollen data (Finsinger et al., 2017; Gosling et al., 2017). Based on a total of 134 recognisable pollen morphotypes for the glacial palynological assemblage of Laguna Pindo, samples N_0_ vary between 40 and 57 morphotypes, N_1_ and N_2_ values rank from 17 up to 24 and from 7 to 18, respectively, and the ratio N_2_/N_0_ ranges between 0.13 and 0.39 (**Table 4**).

## Palaeoecological Interpretation and Discussion

In order to understand the ecological dynamics of the forests recorded in Laguna Pindo, a palaeoclimatic background is needed. Here, independent archives for temperature (Greenland ice core record) and precipitation (Santiago speleothem, Ecuador) reconstructions will be used and placed in a regional context (**Figure 5**). A palaeotemperature record from Greenland is shown instead of the closer record of the Cariaco Basin (offshore Venezuela) as both are equally representatives of the North Atlantic Ocean palaeotemperature (Haug et al., 2001; Deplazes et al., 2013), and the Greenland ice record is expressed directly in temperature degrees (Alley, 2000). Vegetation dynamics will be compared with nearby records when possible and in addition, as Laguna Pindo is located in NW Amazonia at the boundary between Amazon and Andean forests just below the narrow band of cloud forests, comparison with long records retrieved in ecotonal areas from SW and NE Amazonia will be also contemplated.

### Glacial Vegetation at Laguna Pindo

The small catchment size of Laguna Pindo and the persistent abundance of woody taxa (based on pollen and isotopic signals) throughout the glacial period suggest that the Mera region was continuously covered by forest during this period (**Figures 3**, **5**). The large number of pollen taxa found in the glacial record from Laguna Pindo (>130 terrestrial morphotypes) reflects the high biodiversity that characterises western Amazonia and the eastern Andean flank. In this sense, the palynological assemblage of the glacial Pindo forest shows similar values of diversity (N_1_) to modern pollen traps of tropical locations in Bolivia and Ghana (Gosling et al., 2017). It is noteworthy to highlight the high values of diversity obtained in such a present-day closed canopy to the shoreline and small catchment of the lake, an environmental setting prone to collect only evidence from a very local spatial scale (**Figure 1**; Jacobson and Bradshaw, 1981). In addition, it is interesting to observe that despite the homogeneous nature of the richness (N_0_) values along the sequence, the maximum values for all the indices calculated were attained in a single sample, with an estimated age just at the end of the LGM (**Table 4**).

The forest taxa occurring had varied through time based on different climatic and ecological requirements, as forests have been doing since previous glacial-interglacial periods in the tropics and elsewhere (Bush et al., 2004a; Cárdenas et al., 2011a). In this sense, full to late glacial forest in Laguna Pindo was characterised by a mix of taxa that live nowadays in both mid and high elevations (>1000 masl). The continuous occurrence of taxa such as *Alnus*, *Hedyosmum*, *Myrica*, *Podocarpus*, and *Weinmannia* not present in today’s forest (**Table 1**) suggest a colder climate than nowadays, whereas the presence of warm indicators like Arecaceae point to the existence of a no-analogue plant community (Williams and Jackson, 2007). Besides the mixed composition of the glacial forest, Laguna Pindo palynological record is in agreement with previous sequences in another important aspect, the community stability and tolerance during the glacial interval. RoC were calculated and provided very low results of magnitude around ×10^-4^ (**Table 4**). During full glacial conditions, the structure of the forest did not change compared to those observed nowadays, a pre-montane forest. The vegetation shift in composition is due to the coexistence during the ice age of taxa with different climatic requirements in the present-day.

### Dynamics of Vegetation at Laguna Pindo

Laguna Pindo recorded a remarkably continuous lower montane forest cover during the last glaciation, despite several disturbance events or climatic episodes. Disturbance events likely occurred even in the absence of a clear imprint in the sedimentary archive. For instance, a high peak of local fires is located around 43–33 cal kyr BP, or slightly before (with peaks around 52 and 45 cal kyr BP) when expressed in influx values (**Figures 3**, **5**). The occurrence of fires in the wet western Amazonia/eastern Andean flank prior to human arrival were probably caused by volcanic activity (Cárdenas et al., 2011a; Loughlin et al., 2018). However, during this interval no tephras have been observed in Laguna Pindo sequence coeval to the charcoal peak, although volcanic activity is known to have occurred in the region at this time (Keen, 2015; Loughlin et al., 2018). Consequently, a climatic origin for the fires recorded in Laguna Pindo cannot not be entirely ruled out because precipitation levels are thought to have fluctuated in the region throughout the last glacial and both periods with high charcoal in Laguna Pindo record are coeval with drier intervals in Santiago (Mosblech et al., 2012; **Figure 5b**). Regardless of the origin of the fires, the most conspicuous change observed in the vegetation occurred once these stopped, around 27 cal kyr BP also coeval to a drier interval in Santiago (**Figures 5b,d**), which occurred during a time period where the speleothem record was decoupled with insolation (Mosblech et al., 2012).

Based on the trends observed in the vegetation dynamics of the upper section of the sequence, late glacial interval was characterised in Laguna Pindo by a possible gradual change towards the Holocene (**Figures 3**, **5**). In this sense, it can be observed that the shifts in taxa abundance were mostly recorded prior or during LGM, and that the dynamics during the late Glacial were minor, until the record stopped at the beginning of the younger Dryas cold reversal (YD; 12.9–11.7 cal kyr BP). The smoothness observed in the diagram curves could be due to either insensitivity or time-lags of the taxa occurring along the lake shore during this time, or because the climatic change itself was mild at Pindo (i.e., not enough for crossing the tolerance thresholds of the occurring species). The nearby Santiago speleothem record (**Figures 1**, **5b**) suggested a wet late glacial interval (Mosblech et al., 2012) whereas the palaeotemperature reconstruction of the Greenland ice record showed a more stable interval (**Figure 5a**), so it is suggested that both precipitation and temperature could have played a role in the late glacial dynamics of Pindo vegetation. In this sense it can be argued that wet conditions and a stable temperature trend without abrupt extreme values facilitated the late Glacial stability of the vegetation around Pindo, a location that receives >4000 mm yr^-1^ and has a stable annual temperature around 20°C nowadays. Regarding temperature, comparing the palynological groups and the Greenland temperature reconstruction trends (**Figures 5a,c**), it can be observed, that, some changes did occur before the LGM. Thus, during the interval around 30–20 cal kyr BP and especially between Heinrich events H3 and H2, Group 1 (*Miconia*) and 2 (*Hedyosmum*, *Ilex*, and Asteraceae) attained their minimum and maximum values, respectively (**Figure 5c**). *Miconia* is a genus with more than 200 species with different ecological and climatic tolerances in Ecuador and is very abundant in the surroundings of Laguna Pindo in the present-day (**Table 1**; Jørgensen and León-Yánez, 1999). However, some heat-intolerant taxa such as *Alnus*, *Podocarpus*, *Myrica*, and *Hedyosmum* peaked around the LGM core interval (22–18 cal kyr BP; **Figure 3**). Some of the changes in the abundances of *Miconia* and the heat intolerant taxa could be temperature-driven. If so, these shifts in abundances would be in agreement with Lago Consuelo record, a cloud forest location in the southern hemisphere (Peru/Bolivia), which also reported a gradual transition from glacial towards Holocene forests, and was preliminary interpreted in terms of temperature rather than precipitation as the major driver of vegetation changes (Bush et al., 2004b).

With respect to the different taxa sensitivity, the key factor to consider here is the proximity to the occurrence of environmental conditions’ thresholds for the given species. Such ecological proximity could be modified through time due to ecosystem interactions, climatic shifts or feedbacks. Longer records have shown forest stability in more than one glaciation (Bush et al., 2004a). Considering the time scales of glacial versus interglacial duration, it is logical to think that the glacial vegetation was more adapted to cold conditions than to the warm characteristic of the Holocene. Moreover, Bush (2002) hypothesised that as the climate warms, the elevation of cloud formation on the Andean flank would have increased. In Lago Consuelo, the authors suggested a higher stability of glacial forest to dry events as a result of cloud cover formation (Urrego et al., 2010). Therefore, it is proposed here that the forest composition of Pindo during the glaciation was the result of complex environmental interactions, including at least these two factors: (i) the forest taxa had climatic tolerances that include the temperature range (and other parameters like CO_2_ levels) that occurred during the glaciation, and (ii) these lower temperatures of the glacial period facilitated the occurrence of clouds in the wet Laguna Pindo, buffering the effects of a decreased available moisture during the drier intervals. This way, the ecological or climatic threshold was not crossed even during the occurrence of abrupt events resulting in forest stability. The assumption of glacial tolerance range would imply a higher sensitivity to climate change of the forest during interglacials such as the present one. Holocene palaeoecological studies have manifested a high dynamism of plant communities through the entire Amazon basin (e.g., Flantua et al., 2016). Given the current projections on climate change, it is expected that cloud coverage of the eastern Andean flank will continue moving upwards and narrowing as the temperature rises (Bush, 2002; IPCC, 2013). The cloud migration would progressively intensify the vulnerability of large areas of pre-montane and cloud forests, as the buffering effect of the cloud cover moves to higher elevations. The lack of buffer, together with an increased human impact (i.e., increment in land use involving deforestation), would have dramatic consequences for the already threatened biodiversity hotspot of the Ecuadorian eastern Andean flank.

### Dynamics of Glacial Vegetation in the Eastern Andean Flank

The first evidence of non-Andean glacial age forests in tropical South America was from a sedimentary sequence obtained by Paul Colinvaux near the town of Mera, Ecuador (Liu and Colinvaux, 1985) just around 10 km from Laguna Pindo (**Figure 1**). The Mera record was an outcrop exposed by road cutting where a temperature drop of about 4.5°C relative to modern was estimated based on the pollen assemblage found (Liu and Colinvaux, 1985). The estimate of cooling was based on the occurrence of heat-intolerant taxa such as *Alnus*, *Hedyosmum*, and *Podocarpus*. Based on the Mera palynological record, it was suggested that during glacial times tropical forests did not disappear, but were reconfigured and included taxa today only found at much higher elevations. The taxa mixture implied that forests species behaved individually, and not grouped by associations and/or belts, a view those days still not widely accepted outside North America (Whittaker, 1951; van der Hammen, 1974; Davis et al., 1986). Moreover, the glacial forest persistence hypothesis resulting from Mera directly confronted the biogeographic hypothesis of glacial refugia proposed by Haffer (1969) (Colinvaux, 1998; Colinvaux et al., 2000).

The controversial nature of the fossil pollen record from Mera by Liu and Colinvaux (1985) about the structure and composition of the glacial tropical vegetation resulted in several challenges to the interpretation. First of all, the outcrop section contained a large amount of unidentified taxa, as Neotropical palynology was still in its infancy (Colinvaux et al., 1999). Second, the volcanic nature of the inorganic sediment could allow transportation of material from elsewhere, including wood remains of the coniferous tree *Podocarpus* that were found interbedded in Mera’s sediments (Gentry, 1993). Third, this potentially transported material could be much older than the age reported by radiocarbon dating due to contamination and then the record could not be glacial (Heine, 1994). And finally, the uniqueness of a single-site results located within one of the defined refuge areas prevented a general statement of glacial cooling instead of aridity as the main driver of vegetation changes during the ice-age in the tropical forests of South America (Haffer, 1969). Most of these concerns were addressed by the finding of a second glacial record from another outcrop located at San Juan Bosco, just 160 km to the south from Mera, and the setup of modern pollen traps systematic sampling in different locations of Ecuador and Brazil (Bush et al., 1990, 2001; Bush, 1991; Bush and Weng, 2006). San Juan Bosco record showed a mixed assemblage of pollen grains from taxa nowadays living in mid and high elevations as Mera did (Bush et al., 1990). However, the outcrop nature of the record still compromised the potential occurrence of reworked material from volcanic events transportation and hence contamination with older sediments (Heine, 1994).

Subsequent palaeoecological records recovered from lowland tropical sites in South America have supported Liu and Colinvaux’s assertion that taxa currently found at higher, colder elevations coexisted with modern “warm” lowland taxa in the lowlands during the last glacial period (Haberle and Maslin, 1999; van’t Veer et al., 2000; Bush et al., 2004b; Whitney et al., 2011). The closest of the other glacial records to the Mera and San Juan Bosco sites is Vinillos, which is 100 km north of Pindo (**Figure 1**), and is also characterised by a very similar pollen glacial forest assemblage (Loughlin et al., 2018). Finally, the glacial palynological assemblage is not restricted to the last glaciation, as it has been found in previous glacial stadials as well in the region (Cárdenas et al., 2011a). However, all these evidence share the outcrop nature of the record, with the dubious provenance of the sediments, the dating limitations and the potential contamination problems. Laguna Pindo is the first lacustrine record on the westernmost edge of the equatorial Amazonia that registers the last glacial dynamics. By being lacustrine in origin and with such a small catchment, scepticism about the continuity of the sediment, the spatial scale information of the data provided and the volcanic transport contamination can be finally ruled out. Given the volume of the glacial records obtained so far for the Ecuadorian Andean flank, there is a general agreement on the heat intolerant taxa found mixed with current heat tolerant were common and abundant during the last ice age in the eastern Andean flank in western Amazonia.

## Conclusion

Laguna Pindo sequence contains the story of a pre-montane forest in the biodiversity hotspot of the eastern Andean flank of Ecuador during the last glaciation, for the first time obtained from a continuous lacustrine record. The glacial forest of Laguna Pindo has been described as a mix of taxa living nowadays in mid and high elevations. Heat-intolerant taxa including *Podocarpus*, *Alnus*, or *Myrica* showed maximum values around the start of the LGM, replacing the previous dominant taxon, *Miconia.* However, the forest was characterised by stability, in contrast to the Holocene dynamism of Amazon plant communities (Flantua et al., 2016). This stability can be understood in terms of forest structure, being a pre-montane forest during full glacial conditions as it is nowadays, instead of compositional, based on the different climatic and ecological requirements of the occurring species. Wide tolerance ranges to glacial conditions and cloud formation have been proposed as key drivers to maintain Pindo stability. Given the ongoing climate change and the potential scenarios projected, it is more than likely that eventually the clouds that act as buffer of unfavourable climatic events such as droughts will disappear and move upwards, increasing the vulnerability of these tropical forests. Hence, it is essential to adopt management strategies and conservation measures in order to keep the currently desired ecosystem services of this biodiversity hotspot. Finally, the mature or “old-grown” forest concept in the eastern Andean flank or western Amazonia should be revised in order to establish accurate future projections and resilience estimates.

## Author Contributions

EM and WG lead the field work to recover the sediments. HK analysed the charcoal record and created the GIS figure in **Figure 1**. CL provided a vegetation inventory survey. All authors participate in the discussion during the writing of the manuscript, which was lead by EM.

## Conflict of Interest Statement

The authors declare that the research was conducted in the absence of any commercial or financial relationships that could be construed as a potential conflict of interest.
